# Modulation of Cardiometabolic Risk by Vitamin D and K2: Simple Supplementation or Real Drug? Uncovering the Pharmacological Properties

**DOI:** 10.3390/ijms27010298

**Published:** 2025-12-27

**Authors:** Saverio D’Elia, Roberta Bottino, Andreina Carbone, Tiziana Formisano, Massimiliano Orlandi, Simona Sperlongano, Pasquale Castaldo, Daniele Molinari, Alberto Palladino, Mariarosaria Morello, Gisella Titolo, Francesco S. Loffredo, Francesco Natale, Plinio Cirillo, Giovanni Cimmino

**Affiliations:** 1 Cardiology Unit, University Hospital Luigi Vanvitelli, 80138 Naples, Italy; saverio.delia@unicampania.it (S.D.); ro-bottino@hotmail.com (R.B.); andr.carbone@gmail.com (A.C.); tiziana.formisano2019@gmail.com (T.F.); massi.orlandi86@gmail.com (M.O.); simona.sperlongano@unicampania.it (S.S.); pasqualecast@gmail.com (P.C.); daniele.molinari@gmail.com (D.M.); alberto.palladino@policliniconapoli.it (A.P.); 2 Department of Advanced Medical and Surgical Sciences, University of Campania Luigi Vanvitelli, Piazza Luigi Miraglia, 2, 80138 Naples, Italy; gisellatitolo@gmail.com; 3 Department of Public Health, University of Naples “Federico II”, 80131 Naples, Italy; 4 Department of Translational Medical Sciences, Section of Cardiology, University of Campania Luigi Vanvitelli, 80131 Naples, Italy; mariarosaria.morello@gmail.com (M.M.); francesco.loffredo@unicampania.it (F.S.L.); 5 Vanvitelli Cardiology and Intensive Care Unit, Monaldi Hospital, 80131 Naples, Italy; natalefrancesco@hotmail.com; 6 Department of Life Science, Health, and Health Professions, Link Campus University, 00165 Rome, Italy; 7 Department of Advanced Biomedical Sciences, Division of Cardiology, University of Naples “Federico II”, Via Pansini, 5, 80131 Naples, Italy; plinio.cirillo@unina.it

**Keywords:** vitamin D, 25-hydroxyvitamin D, cardiovascular disease, heart failure, hypertension, diabetes, renin–angiotensin–aldosterone system, vitamin K2, bone-vascular crosstalk, supplementation

## Abstract

Vitamin D, traditionally regarded as a nutrient, is increasingly recognized as a pharmacologically active secosteroid with pleiotropic effects extending beyond calcium homeostasis and bone integrity. Together with vitamin K2, it participates in the fine-tuning of mineral metabolism and vascular health, potentially modulating cardiometabolic risk through intertwined endocrine and paracrine pathways. Despite widespread fortification and supplementation, vitamin D deficiency remains a major global health concern, driven by limited sun exposure, obesity, and metabolic dysfunction. Observational and mechanistic studies consistently link low serum 25(OH)D concentrations with hypertension, insulin resistance, heart failure, and increased cardiovascular mortality. At the molecular level, vitamin D exerts pharmacological actions—modulating the renin–angiotensin–aldosterone system, exerting anti-inflammatory and antifibrotic effects, and influencing endothelial and cardiomyocyte signaling. While experimental and epidemiological evidence suggests potential cardiovascular benefits, large randomized controlled trials (RCTs) provide conflicting results, particularly regarding hypertension and heart failure. However, these often-neutral results do not preclude a targeted action. On the contrary, clinical efficacy is strongly dependent on baseline deficiency status and the presence of metabolic cofactors. In this context, high-dose supplementation of Vitamin D, in combination with Vitamin K2 to prevent vascular calcification, elevates the supplement to a genuine pharmacological agent, with a distinct therapeutic potential for modulating cardiometabolic risk in selected patient subgroups. Emerging evidence supports the concept that vitamin D, when appropriately dosed and combined with K2, may act more as a low-potency pharmacological modulator than a simple nutritional supplement. This review synthesizes current mechanistic, observational, and interventional evidence, aiming to clarify whether vitamin D should be reclassified—from a micronutrient to a pharmacologically relevant agent—in cardiometabolic prevention and therapy, proposing a paradigm shift toward personalized and targeted dosing strategies, characteristic of precision pharmacology.

## 1. Introduction

Vitamin D, a pleiotropic secosteroid, exerts physiological effects far beyond its classical roles in calcium homeostasis and skeletal health [1]. Its actions are mediated primarily through the vitamin D receptor (VDR), which is widely expressed across multiple tissues, including endothelial cells, cardiomyocytes, immune cells, and the cardiovascular system [2,3,4,5]. Indeed, a growing number of data indicates its critical involvement in modulating inflammatory processes and influencing cardiovascular well-being [6,7]. This broad tissue distribution underlines the systemic relevance of vitamin D signaling in modulating inflammatory pathways, regulating cytokine release, and influencing cardiovascular homeostasis [6]. Additionally, vitamin D interacts with the renin–angiotensin–aldosterone system, further supporting its role in cardiovascular physiology.

Vitamin D deficiency represents a major global health challenge, affecting nearly one billion individuals worldwide [8]. Definitions of deficiency vary across organizations. The Endocrine Society’s 2011 guidelines are now considered outdated and have been replaced by more recent recommendations (2024 [9]), which no longer aim for a single specific threshold but instead emphasize maintaining sufficient levels for skeletal health while acknowledging limited evidence for extra-skeletal outcomes, including cardiovascular effects The Endocrine Society, previously, the National and International Osteoporosis Foundation, and the American Geriatric Society define deficiency as serum 25-hydroxyvitamin D [25(OH)D] levels <30 ng/mL [10], whereas the National Institutes of Health considers levels <20 ng/mL deficient. Some authorities classify insufficiency as 12–19 ng/mL and severe deficiency as <12 ng/mL [10]. The Institute of Medicine similarly emphasizes a <20 ng/mL threshold, primarily in the context of skeletal health [11,12]. Vitamin D deficiency has been consistently associated with adverse outcomes, including increased susceptibility to cancers, diabetes, hypertension, and cardiovascular disease [13,14]. Epidemiological studies further indicate that populations in regions with higher sun exposure exhibit lower prevalence of deficiency and reduced mortality from chronic disease [15,16]. These findings are reinforced by recent large-scale analyses: for example, a 2023 study in Clinical Nutrition involving nearly 410,000 UK Biobank participants reported that individuals with severe deficiency (<30 nmol/L) had a 10% increased risk of atherosclerotic cardiovascular disease and up to 35% higher risk of cardiovascular mortality [17].

Despite these observations, discrepancies remain between traditional guideline thresholds and emerging evidence. While classical recommendations focus on skeletal outcomes, recent observational data suggest that maintaining serum 25(OH)D concentrations in the 50–70 ng/mL (125–175 nmol/L) range may confer additional cardiovascular benefits [17,18]). However, the extra-skeletal effects of vitamin D remain under active investigation, and randomized controlled trials are required to establish causality and define optimal dosing strategies.

Taken together, these findings provide a compelling rationale for investigating vitamin D supplementation in populations at risk of cardiovascular disease. This review critically evaluates current mechanistic and clinical evidence regarding vitamin D’s influence on cardiovascular risk and mortality, with particular focus on the potential therapeutic benefits of pharmacological supplementation.

It should be noted that the Endocrine Society’s 2024 updated guidelines no longer recommend a fixed serum 25(OH)D threshold for all individuals. Instead, they emphasize individualized sufficiency, primarily aimed at maintaining skeletal health, due to variability in vitamin D metabolism, differences in baseline status, and lack of consistent evidence that higher targets confer additional benefits for all populations [9]. This approach also highlights that any extra-skeletal benefits, including cardiovascular effects, should be considered on a case-by-case basis under medical supervision

## 2. Physiology and Metabolism of Vitamin D

After vitamin D is synthesized in the skin through UVB rays or obtained from our diet, it goes through two important hydroxylation steps. The first one happens in the liver, where the enzyme CYP2R1 transforms vitamin D into 25-hydroxyvitamin D [25(OH)D], which is the primary circulating form and a key indicator of our vitamin D levels. The second hydroxylation occurs in the kidneys, facilitated by CYP27B1, producing the active form, 1,25-dihydroxyvitamin D [1,25(OH)_2_D]. This process is finely tuned by hormones like PTH and fibroblast growth factor 23 (FGF-23), along with negative feedback mechanisms. To prevent excessive hormonal activity, CYP24A1 plays a crucial role in inactivating vitamin D [19].

The diverse effects of vitamin D are largely mediated by its nuclear receptor, the VDR (Vitamin D Receptor), which is part of a larger family of transcription factor receptors. When 1,25(OH)_2_D binds to the VDR, it undergoes a structural change that allows it to pair with the RXR (Retinoid X Receptor). This VDR-RXR complex then attaches to specific DNA sequences known as VDREs (Vitamin D Response Elements), influencing the transcription of about 3–5% of our entire genome [20,21]. Beyond its traditional genomic functions, VDR signaling also taps into epigenetic mechanisms, like histone acetylation through HATs, and gene repression via HDACs and DNMTs. These processes help finely tune gene transcription, especially in areas like inflammation, cell differentiation, and tissue remodeling [22]. Interestingly, the VDR is found in a variety of tissues beyond the usual suspects (like the intestine, bone, kidney, and parathyroids); it is also present in immune cells, endothelial cells, and heart muscle cells, highlighting the widespread influence of vitamin D [20,21,22]. The VDR regulates genes that are crucial for mineral transport and bone remodeling, as well as those involved in both innate and adaptive immune responses, vascular tone regulation, and myocardial fibrosis [22,23]. On a molecular level, the VDR interacts with several intracellular pathways:

- NF-κB: vitamin D inhibits the movement of p65 into the nucleus.
- Wnt/β-catenin plays a role in how osteoblasts differentiate and helps prevent vascular calcification [3].
- TGF-β/Smad is known for its anti-fibrotic effects in cardiovascular tissues [23].
- mTOR/AMPK is involved in energy metabolism and cellular autophagy [22].
- The Renin–Angiotensin–Aldosterone System (RAAS) shows that vitamin D can suppress renin gene transcription, which helps lower blood pressure and offers cardiovascular protection [21,24].

Vitamin D also has some non-genomic effects, which are mediated by:

- Membrane-associated VDR (mVDR)
- PDIA3 (Protein Disulfide Isomerase A3), also referred to as 1,25D3-MARRS

These pathways kickstart quick intracellular signals like PI3K/Akt, MAPK/ERK, and PLC/IP3, which help regulate vascular tone, endothelial function, myocardial contractility, and cell proliferation [22,25].

According to the Institute of Medicine (IOM), vitamin D deficiency is defined as having serum 25-hydroxyvitamin D [25(OH)D] levels below 20 ng/mL (50 nmol/L). Levels above 20 ng/mL are generally seen as sufficient for most people [12]. These guidelines mainly focus on bone health and calcium metabolism, and they are also recognized by the Endocrine Society and the NIH. However, recent studies suggest that higher serum levels, between 50 and 70 ng/mL (125–175 nmol/L), could offer extra cardiovascular benefits. A 2023 study involving nearly 410,000 participants from the UK Biobank found that those with vitamin D deficiency (levels < 30 nmol/L) faced a 10% higher risk of atherosclerotic cardiovascular disease and up to a 35% increase in related mortality [17]. Another review from 2023 indicates that taking at least 2000 IU/day of vitamin D3 might be necessary to reach serum levels above 75 nmol/L, which could be linked to lower cardiovascular mortality. The discrepancy between official guidelines and emerging evidence may reflect the historical focus on bone health rather than cardiovascular outcomes, but the growing body of research indicates a need for updated regulations considering these broader implications. It is, however, essential to emphasize that, while observational studies show promising associations, randomized clinical trials are necessary to confirm causality and optimize dosing strategies [17]. However, it is important to note that while observational studies suggest potential benefits of higher vitamin D levels, randomized controlled trials are necessary to establish causality and determine optimal dosing strategies.

### 2.1. Bone-Vessel Cross-Talk: Vitamin D’s Role in Vascular Health

The idea of bone–vascular crosstalk highlights the intricate relationship between our skeletal and vascular systems, showcasing how they share regulatory mechanisms, hormonal signals, and pathways for mineral metabolism. Cells like osteoblasts, osteoclasts, and vascular smooth muscle cells interact through various mediators, influencing both the remodeling of bones and the calcification of blood vessels. When this balance is disrupted, it can lead to conditions like osteoporosis alongside vascular stiffness or calcification, especially in older adults and those with chronic kidney disease (CKD) [26]. Vitamin D plays a vital role in maintaining vascular health by regulating the metabolism of calcium and phosphate, which are crucial for the mineralization of bones. The bone–vascular connection is influenced by proteins such as osteocalcin and matrix Gla protein (MGP), both of which depend on vitamin K. Osteocalcin, made by osteoblasts, attaches to hydroxyapatite in the bone matrix, while MGP helps prevent vascular calcification by binding to calcium crystals in the walls of blood vessels. Having enough vitamin D is essential for activating these proteins, which helps keep our blood vessels elastic and prevents calcification [27]. Recent research indicates that vitamin K2 supplementation can slow down the progression of coronary artery calcification and reduce arterial stiffness, regardless of vitamin D levels, suggesting that vitamin K2 has a protective effect on vascular health.

Vitamin K comes in two main forms: K1 (phylloquinone) and K2 (menaquinone). While vitamin K1 is mainly involved in blood clotting, vitamin K2 is essential for activating vitamin K–dependent proteins such as osteocalcin and MGP, which are vital for maintaining bone and vascular health. Importantly, while dietary calcium intake appears protective, excessive oral calcium supplementation has been associated with increased coronary artery calcification, highlighting the need for careful consideration of calcium sources. Studies like MESA and Bazarbashi et al. have found a link between oral calcium supplementation and increased coronary artery calcification, while dietary calcium intake seems to offer some protection [28].

This distinction highlights the importance of tailoring calcium supplementation to individual needs and prioritizing dietary sources of calcium whenever possible. Vitamin D plays a crucial role in supporting both bone and vascular health, even without vitamin K2, by boosting the absorption of calcium in the intestines and helping to regulate PTH levels. However, taking high doses of vitamin D without enough K2 could potentially raise the risk of ectopic calcification, especially in people with chronic kidney disease (CKD) or existing atherosclerosis. On a mechanistic level, vitamin D encourages the differentiation and mineralization of osteoblasts, but without carboxylated MGP, the calcium that gets absorbed might end up in the blood vessels.

Vitamin K2 is vital for activating MGP, which is a strong inhibitor of vascular calcification. If there is not enough K2, MGP stays inactive and cannot stop calcium from building up in the arteries, particularly in those with CKD or atherosclerosis.

Taking vitamin D and K2 together seems to enhance bone mineralization while reducing the risk of vascular calcification. Observational studies indicate that K2 can work alongside vitamin D to ensure proper carboxylation of MGP, thus safeguarding the blood vessels while also supporting bone health.

The interplay between vitamin D, K2, and calcium is essential for maintaining both skeletal and cardiovascular well-being:

- Vitamin D boosts calcium absorption, which, in the absence of K2, might heighten the risk of vascular calcification;
- Vitamin K2 serves as a “guardian of the vessels” by activating MGP and preventing unwanted calcium buildup;
- Taking vitamin D and K2 together, along with getting enough calcium from food, seems to provide combined benefits for both the bones and the vascular system.

Emerging research suggests that supplementing with both vitamin D and K2 may be more effective than taking either one alone when it comes to improving bone mineral density and reducing coronary calcification [26,29].

### 2.2. Overview—Formulations and Pharmacologic Agents

Vitamin D for clinical use is available in several molecular forms with distinct pharmacology:

Cholecalciferol (vitamin D_3_)—the most common oral supplement; prohormone that requires 25-hydroxylation (liver) and 1α-hydroxylation (kidney/tissues) to become active [18,30]. Ergocalciferol (vitamin D_2_)—plant-derived form used in some supplements; less potent/long-lasting than D_3_ in raising 25(OH)D [18]. Calcifediol (25-hydroxyvitamin D; 25(OH)D_3_)—the immediate circulating metabolite; oral calcifediol raises serum 25(OH)D faster and more predictably than cholecalciferol and is advantageous in malabsorption, obesity, or when rapid correction is desired. Recent RCTs and meta-analyses show calcifediol is more potent per microgram and yields quicker, linear increases in 25(OH)D [31]. Calcitriol (1,25(OH)_2_D) and alfacalcidol—active forms (or rapidly converted prodrug) used when endogenous 1α-hydroxylation is impaired (advanced CKD, certain hypocalcemic disorders). They bypass regulatory hydroxylation but carry higher risk of hypercalcemia [30] elective VDR agonists (e.g., paricalcitol)—used in CKD/secondary hyperparathyroidism; they have distinct tissue profiles and have been studied for renal and cardiovascular surrogate endpoints (albuminuria, inflammation), with mixed outcome data [32,33].

### 2.3. Pharmacokinetics of Vitamin D and Vitamin K2

Vitamin D and vitamin K2 are both fat-soluble micronutrients that rely on dietary fat and bile acid–mediated micelle formation for intestinal absorption. Vitamin D (D2 and D3) is absorbed in the jejunum and ileum, incorporated into chylomicrons, and transported via the lymphatic system before binding to vitamin D–binding protein (DBP) in circulation. Its pharmacokinetics are shaped by dietary fat content, gastrointestinal integrity, and genetic variants in DBP and related enzymes [33]. Vitamin K2 (menaquinones, especially MK-7) follows a similar absorption pathway but is characterized by a markedly longer half-life—up to 72 h compared with ~1–2 h for vitamin K1—allowing stable serum concentrations and extrahepatic distribution. Although both vitamins share lipid-dependent transport mechanisms, clinical studies demonstrate no significant competition for absorption when administered together, and most trials recommend concurrent intake with meals containing fat to maximize bioavailability [34]. Oral cholecalciferol reaches peak serum concentrations within 12–24 h, with a subsequent distribution phase into adipose tissue and a terminal half-life of approximately two months. Daily administration tends to more stable serum 25(OH)D levels compared with large intermittent boluses, which may lead to greater fluctuations and potentially attenuated physiological responses [35]. Oil-based MK-7 capsules result in faster absorption compared with powder or tablet formulations, though all maintain elevated plasma concentrations for more than one day [36]. In vitro models also suggest that lipid composition can modulate K2 uptake, as medium-chain fatty acids and emulsifiers enhance intestinal absorption.

Vitamin K2, particularly in the menaquinone-7 (MK-7) form, also exhibits fat-dependent absorption but has a longer half-life than vitamin K1, supporting once-daily dosing. Importantly, pharmacokinetic data indicate that vitamin D and K2 do not exhibit significant absorption competition when co-administered, as both are incorporated into chylomicrons but follow distinct metabolic fates. While pharmacokinetic data and clinical trials consistently support concurrent administration of vitamin D and K2 with fat-containing meals, some laboratory-based evidence and theoretical considerations suggest that their absorption kinetics may differ, leading to the proposal—particularly in liquid formulations—that separating intake could optimize bioavailability. However, these claims are not substantiated by randomized controlled trials in humans, and the prevailing evidence indicates no clinically relevant competition when the two vitamins are co-administered [37]. Collectively, these data support the superior bioavailability and longer residence time of MK-7 compared with K1.

Importantly, vitamin D and K2 share fat-dependent uptake pathways via incorporation into chylomicrons but follow distinct metabolic fates, and pharmacokinetic studies have not demonstrated clinically significant competition when co-administered. Clinical trials investigating combined vitamin D and K2 supplementation, such as Knapen et al. [38], indicate potential synergistic effects on bone health, particularly reductions in undercarboxylated osteocalcin, without evidence of impaired absorption. While the overall number of trials is still limited, these results support a possible complementary role of vitamin D and K2 in maintaining bone integrity [39]. While some laboratory findings and theoretical considerations suggest that separation of intake—especially in liquid drop formulations—might optimize absorption kinetics, these claims lack support from randomized human trials. Current evidence therefore favors concurrent administration of vitamin D and K2 with fat-containing meals, without the need for temporal separation.

Recent evidence suggests that daily administration of vitamin D, rather than large intermittent boluses, provides more stable serum 25(OH)D concentrations and may enhance physiological responses, particularly regarding cardiovascular outcomes. Observational and interventional studies indicate that a daily dose of at least 2000 IU of vitamin D3 is often necessary to achieve serum 25(OH)D levels above 75 nmol/L, a threshold associated with reduced cardiovascular disease risk and mortality. While some of these findings have been observed in specific populations, such as lactating women, the data are consistent with broader cohorts and align with the mechanistic rationale supporting daily supplementation. This approach is also compatible with evidence reported by Fassio et al. [40]. Pharmacological features of Vitamin D and Vitamin K1 and K2 are reported in Table 1.

## 3. Role of Vitamin D and CV Diseases

### 3.1. Hypertension

A potential interplay between 25(OH)D levels and cardiovascular homeostasis was initially suggested by links between vitamin D insufficiency and arterial hypertension, especially in pharmacotherapy-resistant cases [41,42]. Multiple pathophysiological pathways have been hypothesized to explain the connection between vitamin D and blood pressure regulation. Foremost among these is an observed interaction between vitamin D and the renin–angiotensin–aldosterone system (RAAS). Experimental studies, for instance, have shown that vitamin D receptor (VDR) knockout mice develop arterial hypertension when compared to control groups, presumably due to heightened plasma renin levels leading to secondary RAAS activation [24]. Additionally, deficient vitamin D activity leads to inadequate calcium levels, secondary hyperparathyroidism, and subsequent arterial hypertension [24]. Also, the vitamin D receptor is notably expressed in endothelial cells, enabling it to modulate vascular tone. This influence is exerted through its control over acetylcholine-responsive systems and the eNOS axis, a mechanism demonstrated by experimental studies using endothelial-specific VDR knockout mice and found to be independent of RAAS activation [43]. Alongside experimental findings, a substantial body of epidemiological and observational research has been conducted, yet these studies have often yielded inconsistent results regarding the relationship between vitamin D and cardiovascular health.

Early epidemiological insights noted a higher incidence of CVD and hypertension in winter and with greater geographical distance from the equator, possibly related to lower sun exposure. The role of race is complex and may involve genetic, environmental, and socioeconomic factors; some studies report variations in vitamin D status among individuals of Black race, but these findings do not directly translate to CVD risk [16,41,44]. Building on such observations, The Third National Health and Nutrition Examination Survey (NHANES III, 1988–1994) revealed an inverse correlation between vitamin D levels and systolic BP in American whites, showing a 20% reduction in systolic BP for concentrations >80 nmol/L [45,46]. Similar findings came from Kim et al.’s cross-sectional analysis of the same NHANES registry (2001–2004), exploring hypovitaminosis D and cardiovascular disease burden [47]. Other observational studies corroborate these data [48,49,50].

In more recent times, the prospective SUN project investigated a cohort of 16,437 Spanish individuals (63.8% female), identifying 2338 new cases of arterial hypertension over a median follow-up period of 12.3 years. The study authors reported a significant inverse relationship between baseline estimated vitamin D levels and the risk of developing hypertension. Notably, participants in the highest quartile of vitamin D levels exhibited a 30% lower risk of arterial hypertension compared to those in the lowest quartile, which suggests a protective role for vitamin D against incident hypertension. These findings remained robust across various demographics and lifestyle factors, including sex, age, BMI, dietary and supplemental vitamin D intake, skin phototype, duration of sun exposure, and physical activity levels [51]. The consistency of these results aligns well with conclusions drawn from prior meta-analyses of observational studies [52,53].

A recent nested case–control study by Wang et al. presents a challenge to this hypothesized association [54]. Authors conducted within the extensive Women’s Health Study (WHS) and the Physicians’ Health Study (PHS) II cohorts, compared 500 incident hypertension cases with 500 age- and race-matched controls drawn from each cohort across both genders [54]. The study’s primary objective was to assess the prospective association between vitamin D biomarkers and hypertension risk, alongside their interrelationship with renin production [54]. The study ultimately found no significant link between baseline levels of vitamin D or parathyroid hormone (PTH) and the subsequent risk of developing hypertension. An initial trend in women (higher vitamin D, lower hypertension) vanished after adjusting for BMI. This persisted across normal and insufficient (<20 ng/mL) vitamin D ranges, with no association in men [54]. The HUNT study, a robust hybrid investigation combining observational analyses within a large cohort and Mendelian randomization, involved approximately 70,000 participants from the HUNT3 (2006–2008) cohort, with a balanced sex distribution (e.g., a subset for specific analyses included 507 men and 559 women) [55]. This study aimed to investigate the relationship between vitamin D, blood pressure, and hypertension risk using a combined approach of traditional observational analysis and Mendelian randomization to infer causality [55]. Observational findings indicated that higher vitamin D levels were inversely correlated with blood pressure and hypertension risk, with each 10 nmol/L increase in vitamin D associated with a modest yet significant reduction. However, the Mendelian randomization analysis did not establish a significant causal association between genetically predicted vitamin D levels and blood pressure or hypertension risk. This suggests that the correlations observed observationally might be attributable to confounding factors or reverse causality, rather than a direct causal effect.

While observational and mechanistic studies propose a potential role for vitamin D in cardiovascular homeostasis, particularly regarding hypertension, large-scale randomized controlled trials (RCTs) have largely failed to demonstrate consistent, significant benefits of vitamin D supplementation on blood pressure (BP) reduction or hypertension prevention. Yet in 2013, Foreman and co-workers conducted a prospective, double-blind, placebo-controlled randomized clinical trial to investigate the effect of vitamin D supplementation on blood pressure in hypertensive African American adults. The study enrolled 283 hypertensive African American adults with a mean baseline 25(OH)D of about 16 ng/mL and randomized them to receive daily doses of 1000 IU, 2000 IU, 4000 IU of vitamin D3, or placebo for 3 months. While all active treatment groups showed significant dose-dependent increases in serum 25(OH)D levels (e.g., ~30 ng/mL in the 4000 IU group), the trial found no significant differences in the change in either systolic or diastolic blood pressure between any vitamin D supplementation group and the placebo group [56]. More recently key RCTs include the VITAL study [57] a large, double-blind, placebo-controlled trial with over 25,000 participants, which found no significant effect of daily vitamin D3 (2000 IU/day) on BP or hypertension incidence. Similarly, the multicenter DO-HEALTH trial in older adults also reported no significant impact of daily vitamin D3 (2000 IU/day) on systolic or diastolic BP after three years [58]. Further, Australia’s D-Health Trial, randomizing over 21,000 participants to monthly vitamin D (60,000 IU) or placebo, also showed no significant effect on BP or hypertension [59]. Even the VITDISH Trial, investigating various doses of vitamin D3 in hypertensive patients, observed no dose-dependent or significant effect on 24 h ambulatory or clinic BP [60]. These individual trial findings are broadly supported by comprehensive meta-analyses of multiple RCTs. For instance, a prominent meta-analysis by Beveridge et al. [61] which included 46 trials for metanalyses purposes (27 of which provided individual patients data sets), found no significant effect of vitamin D supplementation on BP, especially in individuals not severely deficient or already hypertensive. In more recent times Zhang et al. confirmed these conclusions [62]. These meta-analyses consistently underscore the lack of substantial clinical effect across diverse study designs and patient characteristics.

Nevertheless, some meta-analyses present more nuanced findings, suggesting potential benefits in specific contexts. A meta-analysis by Golzarand et al. including 30 RCts reported beneficial effects on BP in subgroup patients such as subjects ≥50 years old on daily vitamin D3 therapy at a dose of >800 IU/day for <6 months and in healthy and hypertensive patients excluding overweight and obese subjects [63]. Witham et al. conducted a metanalysis of 11 RCts suggested that vitamin D supplementation might significantly reduce diastolic BP in individuals with uncontrolled BP (−3.1 mm Hg (95% CI: −5.5–−0.6 [64]. In the same study, a subgroup analysis suggested that supplemental vitamin D had a larger effect on systolic blood pressure (−6.2 mm Hg [95% CI: −12.32–−0.04 mmHg]) than active vitamin D compounds (7 mm Hg [95% CI: −4.8–6.2 mmHg]) [64]. These findings suggest that the therapeutic effect of vitamin D might be restricted to specific patient subgroups, particularly in older hypertensive non obese patients with vitamin D deficiency.

Several methodological limitations contribute to the variability and inconclusiveness of current evidence. Many RCTs, including VITAL [57], DO-HEALTH [58] and D-Health [59] were not primarily designed for BP as a main outcome, potentially lacking sufficient power or adequate follow-up duration for this specific endpoint. Study populations in trials like VITAL and D-Health often include individuals with varying degrees of vitamin D status (from sufficient to severely deficient) and different baseline BP levels, which can obscure potential effects in specific at-risk groups. The heterogeneity in vitamin D dosing regimens (e.g., daily vs. bolus, different doses) and formulations, as seen across various trials including VITDISH [60] as well as the variability in genetic predispositions influencing vitamin D metabolism, further complicates the interpretation and comparability of results. Residual confounding, even in RCTs, and potential issues of adherence to supplementation protocols also pose challenges.

Collectively, despite some conflicting signals in subgroup analyses, these robust RCTs and their meta-analyses predominantly challenge the notion that general vitamin D supplementation offers a direct and widespread clinical benefit for hypertension. Their findings suggest that inverse associations in epidemiological studies may stem from residual confounding or reverse causality, rather than a direct causal effect of vitamin D on blood pressure regulation. This discrepancy highlights the critical importance of randomized evidence in inferring causality and underscores the need for further research, possibly focusing on specific at-risk subgroups, optimal dosing strategies, or alternative vitamin D metabolites, to definitively ascertain any therapeutic role in BP management.

### 3.2. Vitamin D and Diabetes

#### 3.2.1. Pre-Diabetes

Several randomized controlled trials have evaluated whether vitamin D supplementation above levels deemed adequate can reduce the conversion of prediabetes to type 2 diabetes [65,66,67]. The largest and most rigorous study to date, the Vitamin D and Type 2 Diabetes (D2d) trial, randomized 2423 adults with prediabetes to receive cholecalciferol 4000 IU daily or placebo for a median of 2.5 years and found no statistically significant reduction in incident diabetes (hazard ratio 0.88; 95% CI 0.75–1.04) [65]. Subsequent subgroup analyses suggested potential benefit among participants with low baseline 25(OH)D concentrations (<12–20 ng/mL), but these findings were exploratory [67]. Meta-analyses combining D2d and smaller trials have reported modest reductions in diabetes risk and improved reversion to normoglycemia, although heterogeneity among studies and variable baseline vitamin D status limit the certainty of these results [68]. Overall, current evidence does not support routine high-dose vitamin D supplementation for diabetes prevention in individuals with adequate vitamin D status, but it may have value in those who are deficient [69].

Vitamin D insufficiency has been implicated in an elevated risk of both type 1 and type 2 diabetes. Vitamin D receptors are expressed in pancreatic β-cells, and evidence suggests that vitamin D enhances glucose tolerance and reduces insulin resistance [70]. Vitamin D may also exert indirect effects on insulin secretion through calcium-mediated pathways [70]. Additional proposed mechanisms include improved insulin action via upregulation of insulin receptor ex-pression, enhanced glucose transport responsiveness, and modulation of systemic inflammation through direct regulation of cytokine activity [70].

#### 3.2.2. Type 1 Diabetes

Type 1 diabetes is an autoimmune disease characterized by destruction of pancreatic beta-cells. In this setting, Vitamin D may exert protective and immunomodulatory effects, such as down regulating pro-inflammatory and promoting regulatory T-cell activity. Low level of vitamin D has been associated with higher risk of developing type 1 diabetes [71,72]. Some observational studies suggest that vitamin D supplementation in infancy may reduce its incidence, although results are not fully consistent [72,73]. Randomized controlled trials (RCTs) remain limited and inconclusive [74,75,76] with ongoing studies investigating whether supplementation can control the disease progression in high-risk individuals [74,77].

#### 3.2.3. Type 2 Diabetes

Several observational studies have demonstrated an inverse relationship between vitamin D levels and risk of type 2 diabetes, metabolic syndrome, and impaired glucose tolerance. RCTs of vitamin D supplementation have shown heterogeneous results. Some studies report modest improvements in fasting glucose and insulin sensitivity, while others find no significant effect. Benefits appear more pronounced in individuals with baseline vitamin D deficiency or prediabetes.

Recent meta-analyses [78,79,80] suggest that vitamin D supplementation can modestly improve glycemic control in patients with type 2 diabetes, especially those with baseline deficiency and treated with higher doses. However, RCTs in prevention of diabetes show more limited effects [65,81]. Study limitations include heterogeneity in trial design, variable baseline vitamin D status, dosing, duration, and population characteristics.

In conclusion, vitamin D might have a modest but consistent association both in type 1 and type 2 diabetes, delaying the progression of disease and improving glycemic control, respectively. The evidence for prevention of type 2 diabetes is less strong, suggesting a risk reduction. However, Vitamin D supplementation is not recommended only for diabetes prevention. Larger and well-designed RCTs with stratified populations, optimized dosing, and longer durations are needed.

### 3.3. Vitamin D and Heart Failure

In recent decades, a paradoxical vitamin D deficiency has been observed in the Western world, despite high-calorie diets and rising obesity rates. This trend contrasts sharply with the past, when poor nutrition led to Vitamin D deficiency and rickets, even in childhood. This deficiency is now explained by limited exposure to sunlight, related to lifestyle and work (“indoor lifestyle”). This trend has also been observed in patients with HF, both normal weight and overweight/obese [82]. Heart failure is a multifactorial disease with several known causes (coronary artery disease, diabetes, MI, storage diseases), which can occur in different combinations in the same patient. Many new drugs have proven effective in reducing hospitalization rates, mortality, congestion, and quality of life in these patients, but it remains a highly prevalent and fatal disease.

Given these two highly prevalent conditions, it has been hypothesized that vitamin D deficiency may play a role as a pharmacological target in patients with HF. In recent years, studies have demonstrated that vitamin D has pleiotropic effects on the cardiovascular system. Calcitriol acts as an anti-fibrotic and anti-inflammatory agent and as a modulator of the Renin–Angiotensin–Aldosterone System (RAAS). In fact, vitamin D not only increases serum calcium and reduces PTH [83] but also reduces renin, plasma renin activity and aldosterone levels. Low vitamin D levels are associated with higher serum renin levels, and vitamin D treatment in patients with known deficiency significantly reduces plasma renin level [84]. In vitro studies have demonstrated that human endothelial cells from skin biopsies express 1α-hydroxylase and are capable of locally producing 1,25(OH)D, suggesting its autocrine and paracrine role. Cardiomyocytes and fibroblasts from rats evaluated by immunohistochemistry have demonstrated the presence of the vitamin D receptor, whose expression increases in hypertrophied hearts following administration of isoproterenol. [85]. In vitro studies have shown that the addition of vitamin D (1,25 D) or calcitriol to rat mesenchymal cells resulted in a reduced expression of pro-fibrotic factors such as TGFb1, SERPINA1 and Collagen I and III, demonstrating an antifibrotic action. Furthermore, the addition of calcitriol also resulted in an increased expression of metalloproteinases (MMP-8), molecules that degrade collagen or make it more easily digestible by other metalloproteinases, and also of follistatin, an inhibitor of myostatin, an antagonist of muscle cell growth [86]. Lee et al. further published that calcitriol added to cells in vitro resulted in increased mitochondrial ATP production, improving the energy profile of cardiomyocytes [87].

An interesting association study [88], published some years ago, demonstrated that vitamin D deficiency was more common in patients with HF than in healthy controls, albeit with a slight difference (28% vs. 22%), and that vitamin D deficiency was associated with increased mortality in both groups. Refs. [89,90] have demonstrated that mortality and cardiovascular events were more frequent in patients with HF and vitamin D deficiency.

However, a recent meta-analysis of ten studies and 1099 patients [91] did not find evidence that vitamin D supplementation improves heart failure outcomes, including ejection fraction, left ventricular cavity diameters, or BNP levels. It should be noted that these studies are few and often heterogeneous regarding dosage and administration schedule (daily, weekly, or monthly).

In the EVITA Trial [92] daily administration of 4000 IU of vitamin D for 3 years increased the need for mechanical support did not improve heart failure outcomes and was associated with an increased risk of hypercalcemia. The incidence of hypercalcemia was higher in the vitamin D group (6.2%) compared with placebo (3.1%), although this difference was not statistically significant, suggesting careful monitoring of calcium levels may be warranted during supplementation. In this context, better patient selection based on baseline vitamin D, calcium, and PTH levels is likely needed to determine whether vitamin D supplementation has clinical implications in patients with HF.

In summary, vitamin D exerts multiple cardiovascular effects relevant to heart failure, including RAAS modulation, antifibrotic action, and support of cardiomyocyte energetics. Nevertheless, clinical trials remain inconclusive, highlighting the need for targeted studies that account for baseline vitamin D status, calcium, and PTH levels to clarify the potential therapeutic role of supplementation in HF.

**Figure 1:**
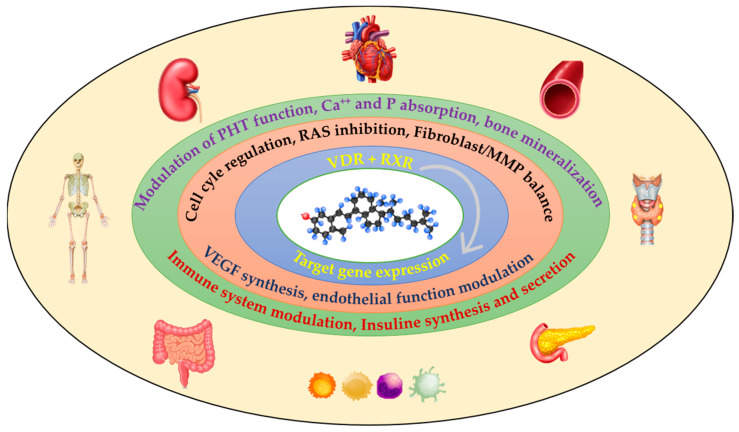
Summary view of Vitamin D function. (PHT: parathormone; Ca: calcium, P: phosphorus; RAS: renin-angiotensin system; MMP: matrix metalloproteinases; VEGF: vascular endothelial grow factor).

A schematic view of Vitamin D function in different organs and apparatus, based on the molecular modulation is provided in Figure 1.

### 3.4. Target Serum Levels of 25(OH)D: Pharmacological Considerations

Defining target 25(OH)D levels is essential not only for bone health but also to potentially modulate extra-skeletal effects, including cardiovascular and metabolic outcomes. These targets guide dosing, formulation choice, and administration frequency.

Defining optimal serum 25-hydroxyvitamin D [25(OH)D] concentrations is crucial for guiding clinical supplementation strategies. Traditional guidelines primarily focus on skeletal health. The Institute of Medicine (IOM) defines vitamin D deficiency as serum 25(OH)D <20 ng/mL (50 nmol/L), with levels ≥20 ng/mL generally considered sufficient to maintain bone mineralization and prevent rickets or osteomalacia [12]. Similarly, the Endocrine Society recommends maintaining serum 25(OH)D levels of 30–50 ng/mL (75–125 nmol/L) to optimize bone health, particularly in populations at risk of osteoporosis or secondary hyperparathyroidism [11]. Emerging evidence suggests that higher 25(OH)D concentrations may confer additional benefits beyond skeletal outcomes, particularly in cardiovascular health. There are currently no randomized controlled trials demonstrating that achieving specific serum 25(OH)D levels reduces cardiovascular risk, and therefore fixed targets for cardiovascular protection are not recommended. Observed associations at higher concentrations (>50–60 ng/mL) do not constitute general recommendations. In fact, while some observational studies in cardiovascular or immunological contexts report potential benefits at higher concentrations (>50–60 ng/mL), these do not constitute general recommendations. Therefore, supplementation strategies should primarily aim to maintain serum levels within the guideline-recommended range, with higher targets considered only in specific clinical scenarios under medical supervision. In fact, observational studies indicate that serum levels above ~50–75 nmol/L are associated with a lower risk of atherosclerotic cardiovascular disease (ASCVD) and cardiovascular mortality [17] but this should be interpreted cautiously and not as a formal target. Achieving these levels often requires daily supplementation of at least 2000 IU of vitamin D_3_, especially in individuals with baseline insufficiency, limited sun exposure, or obesity [40]. It is important to note that while observational data support a potential protective effect of higher vitamin D status on cardiovascular outcomes, randomized controlled trials are needed to establish causality and define optimal dosing. Meanwhile, supplementation strategies should be tailored to the individual’s baseline serum 25(OH)D, comorbidities, and specific clinical goals with consideration of potential extra-skeletal benefits without implying a fixed cardiovascular target, as reflected in Table 2.

Although both vitamin D and vitamin K2 are commercially available as dietary supplements, their use in clinical practice—particularly when co-administered—has characteristics of a pharmacological intervention rather than simple nutrient replacement. First, the regulatory classification of these molecules is dose- and indication-dependent: vitamin D_3_ and calcifediol are approved as medicinal products in several countries for the treatment of deficiency, osteoporosis, and hypoparathyroidism, while vitamin K2 (MK-4) is approved in Japan as a drug for osteoporosis. Second, their combined administration exerts pharmacodynamic actions that exceed nutritional replenishment. Vitamin D increases intestinal calcium absorption, whereas vitamin K2 activates the vitamin-K–dependent proteins osteocalcin and matrix Gla protein (MGP), thereby directing calcium uptake toward bone and preventing ectopic calcification. This coordinated biochemical pathway reflects a drug-like synergistic mechanism, relevant in patients with increased cardiovascular or skeletal risk. Finally, therapeutic co-administration typically follows standardized dosing, clinical indications, and monitoring, consistent with pharmacologic treatment protocols. For these reasons, the combined use of vitamin D and K2 can be considered a targeted therapeutic strategy rather than a generic supplement regimen.

### 3.5. Vitamin K2 and Cardiovascular Health

Vitamin K2 (menaquinone, MK-7) has emerged as a potential modulator of vascular calcification and cardiovascular risk. Several randomized controlled trials (RCTs) have evaluated the effects of MK-7 supplementation on vascular stiffness, coronary artery calcification, and related cardiovascular outcomes. In a 24-month study in elderly men with aortic valve calcification, supplementation with MK-7 combined with vitamin D3 significantly slowed the progression of calcification compared with placebo [95]. In patients with type 2 diabetes and established cardiovascular disease, MK-7 supplementation (360 µg/day for 6 months) improved vascular function and markers of calcification [96]. Long-term studies in patients on hemodialysis demonstrated that MK-7 supplementation attenuates progression of coronary artery calcification [97]. A recent systematic review including 14 RCTs confirmed that vitamin K2 has a favorable effect on markers of vascular calcification, although the clinical cardiovascular outcome data remain limited [98]. In healthy postmenopausal women, daily administration of MK-7 (180 µg for 3 years) improved arterial elasticity and reduced circulating dp-ucMGP, indicating beneficial effects on vascular stiffness and subclinical calcification [38]. Overall, while the number of high-quality trials is still limited, current evidence suggests a potential benefit of MK-7 in reducing vascular calcification, particularly in at-risk populations. Importantly, combination with vitamin D3 has been explored in some trials, providing a mechanistic rationale for synergistic effects on vascular health, but additional studies are warranted to define optimal dosing and target populations. We have summarized the trials in which vitamin K2 was used in cardiovascular settings in Table 3. 

## 4. Conclusions

Vitamin D represents a multifactorial modulator of cardiovascular and skeletal health, with effects on bone–vascular cross-talk and potential benefits on RAAS, cardiac function, and bone metabolism but its role should be carefully evaluated in the context of individual patient characteristics. In specific subgroups it acts as real drug. Optimal administration appears most effective if it is daily, aiming to maintain stable serum 25(OH)D levels, rather than relying on large intermittent boluses. Integration with vitamin K2 is crucial to support proper carboxylation of matrix Gla protein (MGP), thereby enhancing vascular protection while promoting bone mineralization, reflecting the essential bone–vascular cross-talk. Moreover, vitamin D supplementation may be considered alongside specific pharmacologic treatments, including RAAS inhibitors, diuretics, or other cardiovascular drugs, to potentially optimize outcomes in at-risk populations. Careful monitoring of vitamin D, calcium, and PTH levels is recommended to personalize therapy, maximize benefits, and minimize risks such as hypercalcemia or ectopic calcification. However, administration should not be liberal but always under medical supervision, considering the patient’s clinical status and potential drug interactions, to prevent possible toxicities such as hypercalcemia or ectopic calcifications.

## Figures and Tables

**Table 1:** Pharmacological features of Vitamin D and Vitamin K1 and K2.

	Vitamin D3 (Cholecalciferol)	Vitamin K1 (Phylloquinone)	Vitamin K2 (Menaquinone-7, MK-7)
**Parameter**			
**Form**	Secosteroid	Phylloquinone	Menaquinone-7
**Route**	Oral	Oral	Oral
**Fat-dependent absorption**	Yes	Yes	Yes
**Tmax**	12–24 h	1–2 h	4–6 h
**Half-life**	~60 days (25[OH]D)	1–3 h	48–72 h
**Bioavailability**	Increased with fat-containing meal; distributed into adipose tissue	Increased with fat; rapid clearance	Increased with fat; long circulation, supports once-daily dosing
**Absorption competition**	None clinically relevant with K2	None clinically relevant with D3	None clinically relevant with D3; theoretical considerations in lab studies suggest slight kinetic differences
**Clinical notes**	Daily dosing stabilizes 25(OH)D; large bolus may cause fluctuations	Short half-life limits affect duration; primarily for hepatic functions	Long half-life allows once-daily dosing; synergistic with vitamin D in bone and vascular health

**Table 2:** Suggested targets in the population at risk.

Indication	25(OH)D Target (ng/mL)	25(OH)D Target (nmol/L)	Suggested Dose/ Formulation	Notes/References
Osteoporosis/Bone Health	20–30	50–75	Oral vitamin D3 800–2000 IU/day; daily or weekly supplementation depending on baseline status	Sufficient to prevent fractures and osteomalacia; [1]
Cardiovascular Risk Prevention Observed range associated with lower CVD risk (observational evidence)	Observed range 30–50 (not a formal target)	75–125	Oral vitamin D3 2000–4000 IU/day; calcifediol may be preferred for rapid correction in severe deficiency	Observational studies and meta-analyses suggest reduced ASCVD risk and mortality at ≥75 nmol/L, but no RCT demonstrates a cardiovascular protective effect Higher levels (>50–60 ng/mL) may be considered only in selected clinical contexts under medical supervision [93,94]. Recent Endocrine Society 2024 guidelines no longer recommend a fixed threshold; focus is on individualized sufficiency, primarily for skeletal health [9]
At-Risk Populations (elderly, CKD, obesity, malabsorption)	30–50	75–125	Vitamin D3 2000–4000 IU/day; consider calcifediol in malabsorption or obesity; co-supplementation with vitamin K2 (MK-7 100–200 μg/day)	Higher deficiency risk and blunted response; goal to optimize extra-skeletal benefits
Toxicity/Hypervitaminosis D	>100	>250	Avoid high-dose supplementation without monitoring	Risk of hypercalcemia, ectopic calcification, nephrolithiasis

**Table 3:** Randomized controlled trials evaluating Vitamin K2 (menaquinone-7).

Study (Year)	Population/Design	Intervention (Dose, Duration)	Comparator	Main Findings
Diederichsen et al., 2022 (AVC Trial) [95]	365 elderly men with aortic valve calcification, double-blind RCT	MK-7 720 µg/day + vitamin D3 for 24 months	Placebo	Slower progression of aortic valve calcification and reduced dp-ucMGP levels vs. placebo
Zwakenberg et al., 2019 [96]	68 patients with T2DM + CVD, randomized, 6 months	MK-7 360 µg/day	Placebo	Improved arterial stiffness and reduction in uncarboxylated MGP; trend toward reduced calcification
RenaKvit Study, 2021 [97]	190 patients on chronic hemodialysis, double-blind RCT	MK-7 360 µg/day for 24 months	Placebo	Attenuation of coronary artery calcification progression; safe and well tolerated
Knapen et al., 2015 (Rotterdam Study Extension) [38]	244 healthy postmenopausal women, 3-year trial	MK-7 180 µg/day	Placebo	Improved arterial elasticity and decreased uncarboxylated MGP
Frontiers Systematic Review, 2023 [98]	Systematic review of 14 RCTs	Various MK-7 or MK-4 formulations	—	Consistent reduction in dp-ucMGP; modest but favorable effects on vascular calcification; limited outcome data

## Data Availability

No new data were created or analyzed in this study. Data sharing is not applicable to this article.
